# Development and external validation of a predictive scoring system associated with metastasis of T1‐2 colorectal tumors to lymph nodes

**DOI:** 10.1002/ctm2.30

**Published:** 2020-04-30

**Authors:** Shaobo Mo, Zheng Zhou, Weixing Dai, Wenqiang Xiang, Lingyu Han, Long Zhang, Renjie Wang, Sanjun Cai, Qingguo Li, Guoxiang Cai

**Affiliations:** ^1^ Department of Colorectal Surgery Fudan University Shanghai Cancer Center Shanghai China; ^2^ Department of Oncology Shanghai Medical College Fudan University Shanghai China; ^3^ Department of Cancer Institute Fudan University Shanghai Cancer Center Fudan University Shanghai China

**Keywords:** colorectal cancer, lymph node metastasis, nomogram, T1‐2

## Abstract

**Background:**

It is critical for determining the optimum therapeutic solutions for T1‐2 colorectal cancer (CRC) to accurately predict lymph node metastasis (LNM) status. The purpose of the present study is to establish and verify a nomogram to predict LNM status in T1‐2 CRCs.

**Methods:**

A total of 16 600 T1‐2 CRC patients were enrolled and classified into the training, internal validation, and external validation cohorts. The independent predictive parameters were determined by univariate and multivariate analyses to develop a nomogram to predict the probability of LNM status. The calibration curve, the area under the receiver operating characteristic curve (AUROC), and decision curve analysis (DCA) were used to evaluate the performance of the nomogram, and an external verification cohort was to verify the applicability of the nomogram.

**Results:**

Seven independent predictors of LNM in T1‐2 CRC were identified in the multivariable analysis, including age, tumor site, tumor grade, perineural invasion, preoperative carcinoembryonic antigen, clinical assessment of LNM, and T stage. A nomogram incorporating the seven predictors was constructed. The nomogram yielded good discrimination and calibration, with AUROCs of 0.72 (95% confidence interval [CI]: 0.70‐0.75), 0.70 (95% CI: 0.67‐0.74), and 0.74 (95% CI: 0.71‐0.79) in the training, internal validation, and external validation cohorts, respectively. DCA showed that the predictive scoring system had high clinical application value.

**Conclusions:**

We proposed a novel predictive model for LNM in T1‐2 CRC patients to assist physicians in making treatment decisions. The nomogram is advantageous for tailoring therapy in T1‐2 CRC patients.

**Abbreviations**
AD: adenocarcinoma
AUC: the area under the curve; ROC, receiver operating characteristic
CEA: carcinoembryonic antigen
CI: confidence interval
cLNM: clinical assessment of lymph node metastasis
CRC: colorectal cancer
CSS: cause‐specific survival; DFS, disease‐free survival
CT: chemotherapy; AUROC, area under receiver operating characteristic curve
DCA: decision curve analysis
FUSCC: Fudan University Shanghai Cancer Center
HR: hazard ratio
IQR: interquartile range
LNM: lymph node metastasis
MAD: mucinous adenocarcinoma
OS: overall survival
SD: standard deviation
SEER: surveillance, epidemiology, and end results
SRCC: signet ring cell carcinoma
TNM: tumor‐node‐metastasis

## 1 BACKGROUND

Ranking as the third leading cause of death among all malignancies, the incidence of colorectal cancer (CRC) has continued to rise over the past three decades worldwide, which has made CRC a critical problem for public health.^1^ The status of lymph node metastasis (LNM) provides valuable information for the selection of treatment strategies for CRC, which have important effects on the prognosis of CRC patients.^2^ According to the tumor stage defined by the seventh TNM staging system, the incidence of LNM in patients with stage I CRC, which contains T1 and T2, was between 8.4% and 23.5%, leading to higher TNM stages and mortality.^3^, ^4^ The 5‐year survival rate of T1‐2 CRC patients with LNM positivity (stage III CRC) is less than 70%, while the 5‐year survival rate of T1‐2 CRC patients without LNM (stage I CRC) is more than 90%.^5^ Based on information from the diagnostic workup, including physical examination, imaging, diagnostic lymph node biopsy, and exploratory surgery without resection, the clinical assessment of LNM (cLNM) status was slightly different from the actual LNM status. Thus, it is important to predict LNM status in patients with T1‐2 CRC in order to select more reasonable surgical and chemotherapy regimens to improve survival.

Cases confined to the muscularis propria (T1 and T2) without LNM or distant metastasis are generally considered early neoplastic lesions that can be potentially cured by complete resection of the tumor.^6^ When LNM occurs in stage T1‐2 CRC patients, the early stage of the disease will progress to a later stage, requiring radical surgery and lymph node dissection, followed by 3 or 6 months of postoperative adjuvant chemotherapy. With the growing performance of endoscopy screening procedures and the rapid development of endoscopy technology, the prevalence of endoscopic resection in early CRC has recently been expanding. The endoscopic resection of early CRC lesions should be performed selectively, because the occurrence of LNM in CRCs can affect the results of endoscopic resection. Predicting LNM status in T1‐2 CRC patients is critical for determining whether patients should undergo additional radical surgery.^7^, ^8^ Several clinicopathological parameters, such as histological type, preoperative carcinoembryonic antigen (pre‐CEA) levels, depth of submucosal infiltration, and perineural invasion are predictors of LNM.^9^ According to the current guidelines, the LNM status of CRC patients with stage T1 disease is evaluated in a dichotomous manner in routine clinical practice, including low or high risk. If the patient has any risk features after endoscopic resection, the T1 CRC patient falls into high risk, and additional radical surgery is recommended to perform regional lymphadenectomy. However, due to the low sensitivity and specificity of these detection and classification patterns, they might result in overtreatment and lead to a decrease in the quality of life of CRC patients, especially in low rectal cancer patients requiring anal resection. Therefore, a new simple model is needed to help clinicians predict the risk of LNM in T1‐2 CRC patients, to reduce the possibility of current overtreatment, and to more accurately identify LNM in T1‐2 CRC patients who need additional lymphadenectomy and postoperative adjuvant therapy.

The Surveillance, Epidemiology, and End Results (SEER) database functionally offers a profusion of integral clinical and pathological information for various cancers that cover ∼28% of the American population. In this study, information on CRC patients was gathered from the SEER database and Fudan University Shanghai Cancer Center (FUSCC) cohorts. First, we divided the cases in the SEER database into a training set and an internal validation set. Then, we developed an intuitive and comprehensive nomogram to predict the probability of LNM in T1‐2 CRC patients in the training set, evaluated the model's efficacy in the internal validation set, and further validated the model's predictive power in the FUSCC external validation set.

## 2 METHODS

### 2.1 Ethics statement

The Ethical Committee and Institutional Review Board of the Fudan University Shanghai Cancer Center reviewed and approved this study protocol. All patients provided written informed consent.

### 2.2 Patient selection

**FIGURE 1:**
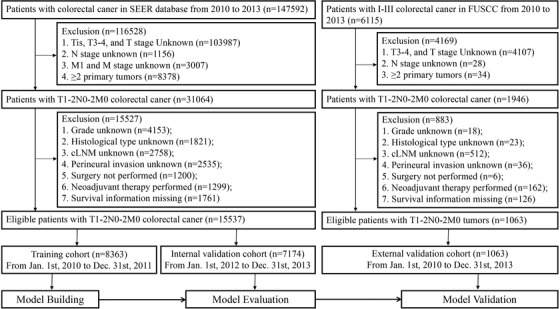
Recruitment pathway of eligible T1‐2 CRC patients in this study

In this study, a total of 15 537 eligible patients from the SEER dataset and 1063 patients from the FUSCC cohort with T1‐2 CRC were acquired. The detailed inclusion and exclusion criteria of patients are shown in Figure 1. All CRC patients treated with radical resection between January 1, 2010, and December 31, 2013, were evaluated for inclusion and retrospective analysis. Patients with non‐CRC, Tis, T3, T4, or unknown TNM stage cancers and those suffering from at least two malignant tumors were excluded. Eleven variables were extracted in this study, including sex, age, pre‐CEA level, pathological grade, tumor size, primary site, perineural invasion, histological type, cLNM, chemotherapy (CT), and T stage. LNM status was determined by the pathologists after the patient underwent radical surgery. All clinicopathological factors were assessed according to the *AJCC Cancer Staging Manual*, 7th Edition.^10^ The specific details of each clinicopathological factor are as follows: (1) Primary site: Right‐sided CRCs included tumors in the transverse colon, hepatic flexure of the colon, ascending colon and cecum; and left‐sided CRCs included tumors in the rectum, rectosigmoid junction, sigmoid colon, descending colon, and splenic flexure of the colon. (2) Histological type: Histological type of CRC patients was identified by the *International Classification of Diseases for Oncology*, 3rd Edition (ICD‐O‐3). According to ICD‐O‐3 oncology codes, three histological subtypes of CRCs were classified as follows: adenocarcinoma (8010, 8020, 8140–8144, 8210, 8211, 8255, 8260–8263, 8310, 8440, 8460, 8550, 8560), mucinous adenocarcinoma (8470‐8472, 8480, 8481), and signet ring cell adenocarcinoma (8490). (3) Pathological grade: Well‐differentiated tumor was classified as grade I; moderately differentiated tumor was divided into grade II; poorly differentiated and undifferentiated tumors were identified as grade III and grade IV, respectively. (4) Pre‐CEA level: In the SEER cohort, the pre‐CEA level refers to “CS Site‐Specific Factor 1,″ where “Code 010″ means “Positive,” “Code 020″ means “Negative,” and other codes mean “Other.” In the FUSCC cohort, pre‐CEA levels were determined by an electrochemiluminescence immunoassay using a Roche Cobas e601 immunoassay analyzer (Roche Diagnostics, Mannheim, Germany). The normal upper limit of pre‐CEA was adopted as 5.2 ng/mL. (5) cLNM: The clinical assessment of LNM status was based on information from the diagnostic workup, including physical examination, imaging, diagnostic lymph node biopsy, and exploratory surgery without resection. Patients’ survival information was quantified by cause‐specific survival (CSS), disease‐free survival (DFS), and overall survival (OS), and those with missing survival information were excluded. Finally, 15 537 patients with T1‐2 CRC from the SEER database were divided into training and internal validation sets for model building and evaluation. Eligible patients from the FUSCC cohort were used as the external validation cohort for model validation.

### 2.3 Construction and validation of the nomogram

Univariable and multivariable logistic regression analyses were used to calculate and validate the effect of variables in the training, internal validation, and external validation cohorts. The variables with *P* < .05 in the univariate model were used in the multivariate logistic regression analysis. The measure of the effect of each variable on LNM was presented as an odds ratio (OR) to identify independent risk factors. Based on the multivariable logistic regression analysis results, a nomogram integrating clinicopathological parameters with cLNM was formulated. The overall points for each patient in the training, internal validation and external validation cohorts were calculated using the established nomogram, after which a logistic regression analysis of the entire cohort was carried out using the overall points as a parameter. The sensitivity, specificity, positive predictive value (PPV), and negative predictive value (NPV) of the nomogram were calculated.

### 2.4 The calibration curve and area under the receiver operating characteristic curve

The calibration of the nomogram was evaluated by the Hosmer‐Lemeshow test and displayed in the form of the calibration curve. The accuracy of nomogram is displayed in the form of ROC curve, and the discriminative ability of the nomogram to predict LNM status in T1‐2 CRC is quantitatively expressed by the area under the receiver operating characteristic curve (AUROC).

### 2.5 Clinical usefulness

Decision curve analysis (DCA) is a new method to evaluate the potential clinical value of a risk prediction model, which can directly reflect the potential benefits of the new model once applied in clinical practice^11^; thus, the DCA method was performed to compare the clinical consequences of the predictive nomogram in the current research.

### 2.6 Survival analyses

Survival curves for different groups (LNM negative: T1‐2 CRC patients without lymph node metastases; LNM positive: T1‐2 CRC patients with lymph node metastases; LNM positive without CT: LNM positive patients without adjuvant chemotherapy; and LNM positive with CT: LNM positive patients with adjuvant chemotherapy) were plotted using the Kaplan‐Meier method and the differences among them were compared using the log‐rank test. CSS in the SEER dataset was defined as the time of the diagnosis of CRC to the time when CRC cause‐specific death occurred. Death from CRC was considered an event, and death from other causes or survival at the end of the follow‐up was censored.

### 2.7 Statistical analysis

R software (version 3.6.1, http://www.r-project.org) was used for all statistical analyses. The R statistical packages “rms,” “barplot,” “survival,” “Hmisc,” “MASS,” and “pROC” were used to plot the distribution of risk scores and LNM, plot calibration, and logistic ROC curves, build a nomogram, and draw Kaplan‐Meier curves, while “rmda” was used to draw the DCA curves and “forestplot” was used to draw the forest plot. Continuous variables were presented as the mean ± SD, and the difference was analyzed using one‐way ANOVA or Student's *t*‐test. The differences in categorical variables were analyzed by Fisher's exact test or a two‐tailed χ^2^ test. All statistical tests were two‐sided, and *P*‐values < .05 were regarded as statistically significant.

## 3 RESULTS

### 3.1 Patients’ clinicopathological characteristics and prognosis

A total of 16 600 patients with T1 and T2 CRC were retrospectively enrolled from the SEER (n = 15 537) and FUSCC (n = 1063) cohorts. The LNM rate was 14.2% (9.6% for T1 stage and 18.9% for T2 stage) and 20.1% (9.8% for T1 stage and 23.4% for T2 stage) for all T1‐2 CRC patients in the SEER and FUSCC cohorts, respectively. The patients’ clinicopathological characteristics according to LNM status and a comparison of the clinicopathological factors in the training, internal validation, and external validation cohorts are listed in Table 1 and Supporting Information Table 1, respectively. The median age of T1‐2 CRC patients was 66.0 years old (interquartile range (IQR), 56.0‐76.0) and 60.0 years old (IQR, 52.0‐67.0) in the SEER and FUSCC cohorts, respectively. In both the SEER and FUSCC cohorts, compared with patients without LNM, T1‐2 patients with LNM were more likely to be younger than 60, have poor tumor grade, be pre‐CEA positive, have more perineural invasion, have a higher frequency of T2 status, and have tumors localized more commonly in the left‐sided colon. Moreover, the cLNM positive rate was 12.0% for all patients with LNM compared with 0.8% for all patients without LNM. A total of 11.8% (68.4% for LNM positive) and 19.6% (84.6% for LNM positive) of patients underwent adjuvant chemotherapy in the SEER and FUSCC cohorts, respectively.

**TABLE 1:** Clinicopathological characteristics of patients in the training, internal validation and external validation cohorts (N(%))

Characteristics	SEER						FUSCC		
	Training cohort			Internal validation cohort			External validation cohort		
	LNM (–)	LNM (+)	*P* value	LNM (–)	LNM (+)	*P* value	LNM (–)	LNM (+)	*P* value
	N = 7173	N = 1190		N = 6154	N = 1020		N = 849	N = 214	
Age			*P* < .001			*P* < .001			.002
< 60	2198(30.6)	477(40.1)		1876(30.5)	402(39.4)		401(47.2)	127(59.3)	
≥60	4975(69.4)	713(59.9)		4278(69.5)	618(60.6)		448(52.8)	87(40.7)	
Mean±SD	66.5±12.8	63.7±13.4	*P* < .001	66.4±12.5	63.1±12.5	*P* < .001	59.7±11.4	57.4±11.4	.009
Median (IQR)	67(57‐76)	63(54‐74)		67(57‐76)	63(54‐72)		60(53‐68)	58(50‐64)	
Gender			.944			.303			.566
Female	3498(48.8)	579(48.7)		3012(48.9)	517(50.7)		394(46.4)	104(48.6)	
Male	3675(51.2)	611(51.3)		3142(51.1)	503(49.3)		455(53.6)	110(51.4)	
Primary site			.002			.001			.010
Right	3018(42.1)	445(37.4)		2621(42.6)	378(37.1)		134(15.8)	19(8.9)	
Left	4155(57.9)	745(62.6)		3533(57.4)	642(62.9)		715(84.2)	195(91.1)	
Grade			*P* < .001			*P* < .001			*P* < .001
I	1250(17.4)	94(7.9)		1006(16.3)	111(10.9)		158(18.6)	16(7.5)	
II	5419(75.5)	872(73.3)		4672(75.9)	758(74.3)		605(71.3)	156(72.9)	
III and IV	504(7.1)	224(18.8)		476(7.7)	151(14.8)		86(10.1)	42(19.6)	
Histological type			.003			.385			.658
AD	6863(95.7)	1115(93.7)		5895(95.8)	971(95.2)		789(92.9)	197(92.1)	
MAD and SRCC	310(4.3)	75(6.3)		259(4.2)	49(4.8)		60(7.1)	17(7.9)	
Tumor Size			.102			.642			.247
< 4	4531(63.2)	781(65.6)		4075(66.2)	683(67.0)		513(60.4)	120(56.1)	
≥4	2642(36.8)	409(34.4)		2079(33.8)	337(33.0)		336(39.6)	94(43.9)	
Perineural invasion			*P* < .001			*P* < .001			*P* < .001
No	7066(98.5)	1116(93.8)		6063(98.5)	958(93.9)		825(97.2)	193(90.2)	
Yes	107(1.5)	74(6.2)		91(1.5)	62(6.1)		24(2.8)	21(9.8)	
Pre‐CEA			*P* < .001			*P* < .001			*P* < .001
Negative	3027(42.2)	565(47.5)		2578(41.9)	482(47.3)		630(74.2)	112(52.3)	
Positive	649(9.0)	173(14.5)		596(9.7)	153(15.0)		219(25.8)	102(47.7)	
Other	3497(48.8)	452(38.0)		2980(48.4)	385(37.7)		—	—	
cLNM			*P* < .001			*P* < .001			*P* < .001
Negative	7128(99.4)	1040(87.4)		6101(99.1)	902(88.4)		837(98.6)	192(89.7)	
Positive	45(0.6)	150(12.6)		53(0.9)	118(11.6)		12(1.4)	22(10.3)	
Adjuvant CT			*P* < .001			*P* < .001			*P* < .001
No	6994(97.5)	409(34.4)		6016(97.8)	289(28.3)		822(96.8)	33(15.4)	
Yes	179(2.5)	781(65.6)		138(2.2)	731(71.7)		27(3.2)	181(84.6)	
T stage			*P* < .001			*P* < .001			*P* < .001
T1	3763(52.5)	394(33.1)		3274(53.2)	349(34.2)		231(27.2)	25(11.7)	
T2	3410(47.5)	796(66.9)		2880(46.8)	671(65.8)		618(72.8)	189(88.3)	

Abbreviations: SEER, Surveillance, Epidemiology, and End Results; FUSCC, Fudan University Shanghai Cancer Center; LNM, lymph node metastasis; cLNM, clinical assessment of lymph node metastasis; SD, standard deviation; IQR, interquartile range; CEA, carcinoembryonic antigen; AD, adenocarcinoma; MAD, mucinous adenocarcinoma; SRCC, signet‐ring cell carcinoma; CT, chemotherapy

In the SEER database, the median follow‐up period was 24 ± 12 months (ranging from 3 to 47 months). The estimated 3‐year CSS rate was 90.7% for all patients (Supporting Information Figure 1A). In the FUSCC cohort, 80 patients experienced recurrence, while the other 983 patients were still free of disease at the last follow‐up, with a median follow‐up time of 55.2 months (ranging from 4 to 82 months). The 5‐year DFS rate was 87.1%, and the 5‐year OS rate was 92.4% for all patients (Supporting Information Figure 1B,C). In the SEER cohort, the LNM‐negative group had a higher CSS of 91.0% for 3‐year CSS compared with 89.9% in the LNM‐positive group (*P* = .010, Supporting Information Figure 2A). Similar findings were confirmed in the FUSCC cohort, with a 5‐year DFS of 89.6% versus 78.2% (*P* = .001, Supporting Information Figure 2C) and a 5‐year OS of 95.1% versus 82.2% (*P* < .001, Supporting Information Figure 2E) for the LNM‐negative group and LNM‐positive group.

In view of the adjuvant chemotherapy (CT) status for the LNM‐positive group, we divided the LNM‐positive group into subgroups of LNM‐positive with CT and LNM‐positive without CT. Kaplan‐Meier analysis revealed that LNM‐positive patients with CT in the SEER cohort had the best 3‐year CSS compared with the LNM‐negative group and LNM‐positive patients without CT (93.7% vs 91.0% vs 82.0%, *P* < .001, Supporting Information Figure 2B). Moreover, the 5‐year DFS rates of the LNM‐negative group, LNM‐positive with CT group, and LNM‐positive without CT group were 89.6%, 79.6%, and 75.7%, respectively, in the FUSCC cohort (*P* < .001, Supporting Information Figure 2D). Furthermore, the Kaplan‐Meier analysis revealed no statistical significance between the LNM‐positive with CT and LNM‐positive without CT groups, while both of them had worse 5‐year OS rates than the LNM‐negative group (85.4% and 87.9% vs 95.1%, *P* < .001, Supporting Information Figure 2F). The above results showed that accurate assessment of LNM status in T1‐2 CRC patients to formulate specific therapeutic regimens (surgery or surgery plus adjuvant CT) had a crucial impact on the prognosis of patients.

### 3.2 Independent predictive features in T1‐2 CRC patients and construction of the nomogram

**FIGURE 2:**
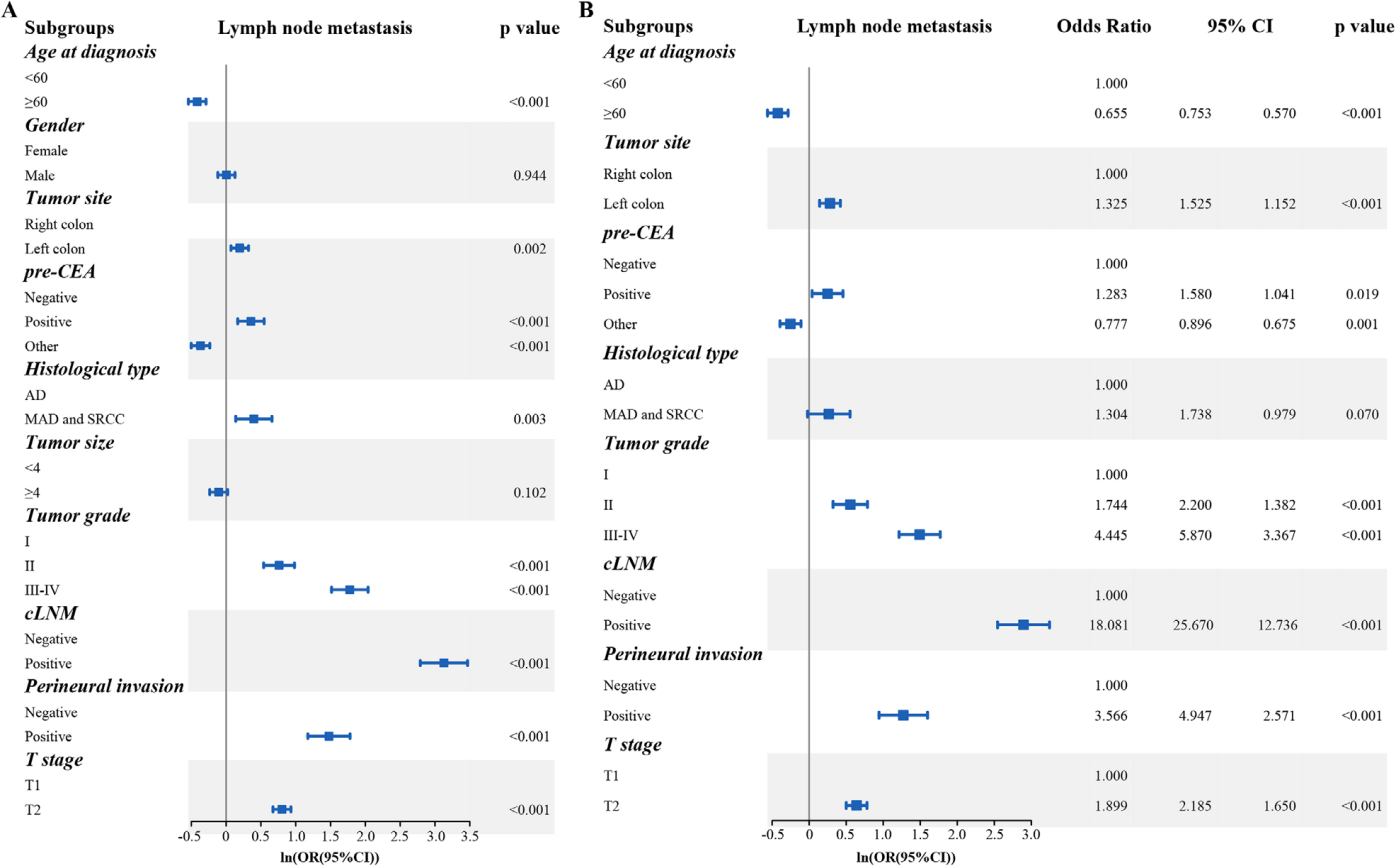
Univariable **(A)** and multivariable **(B)** logistic regression models were used to analyze associations of patients’ characteristics against LNM status in T1‐2 CRC patients. Hazard ratios (HRs) and 95% confdence intervals (CIs) were estimated and summarized with forest plots

**FIGURE 3:**
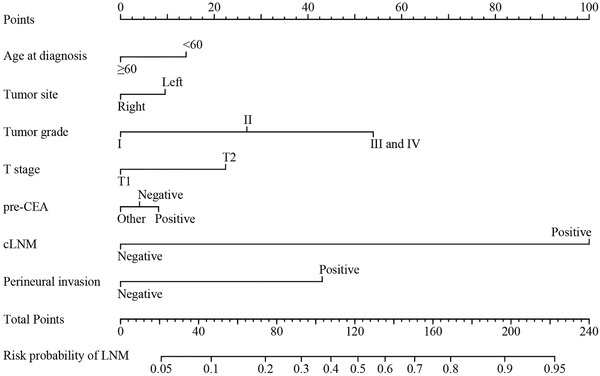
Newly developed nomogram for predicting LNM in T1‐2 CRC patients

Based on the univariate logistic regression analysis results in the training cohort, eight factors, including age at diagnosis, pre‐CEA level, primary tumor site, histological type, pathological grade, perineural invasion, T stage, and cLNM, were linked to LNM status (Table 2 and Figure 2A). In multivariate logistic regression analysis, seven variables, including age at diagnosis, pre‐CEA level, primary tumor site, pathological grade, perineural invasion, T stage, and cLNM, were determined as independent predictive parameters of LNM status in T1‐2 CRC patients (Table 3 and Figure 2B). Multivariable logistic regression analysis was used to identified the significant features associated with LNM status, based on which the nomogram for LNM was developed and shown in Figure 3. The risk score corresponding to each variable can be obtained by the top ruler, and the probability of LNM can be calculated by superposing the risk score of each variable to the bottom ruler. Detailed point assignments and predictive scores for each variable in the nomogram model are listed in Supporting Information Table 2.

**TABLE 2:** Univariate logistic regression model in the training, internal validation and external validation cohorts

		Univariate logistic regression					
		Training cohort		Internal validation cohort		External validation cohort	
Subgroups		OR (95% CI)	*P* value	OR (95% CI)	*P* value	OR (95% CI)	*P* value
Age at diagnosis	<60	1		1		1	
	≥60	0.660(0.582‐0.749)	*P* < .001	0.674 (0.588‐0.773)	*P* < .001	0.613(0.452‐0.831)	.002
Gender	Female	1		1		1	
	Male	1.004(0.888‐1.136)	.944	0.933 (0.817‐1.065)	.303	0.916(0.678‐1.236)	.566
Tumor site	Right colon	1		1		1	
	Left colon	1.216(1.072‐1.380)	.002	1.260 (1.099‐1.445)	.001	1.923(1.160‐3.190)	.011
pre‐CEA	Negative	1		1		1	
	Positive	1.428(1.181‐1.727)	*P* < .001	1.373 (1.121‐1.681)	.002	2.620(1.923‐3.569)	*P* < .001
	Other	0.692(0.606‐0.791)	*P* < .001	0.691 (0.598‐0.798)	*P* < .001	—	—
Histological type	AD	1		1		1	
	MAD and SRCC	1.489(1.148‐1.931)	.003	1.149 (0.840‐1.570)	.385	1.135(0.648‐1.988)	.659
Tumor size	<4	1		1		1	
	≥4	0.898(0.790‐1.022)	.102	0.967 (0.840‐1.113)	.642	1.196(0.883‐1.619)	.247
Tumor grade	I	1		1		1	
	II	2.140(1.715‐2.670)	*P* < .001	1.470 (1.191‐1.815)	*P* < .001	2.546(1.479‐4.385)	.001
	III‐IV	5.910(4.547‐7.681)	*P* < .001	2.875 (2.199‐3.759)	*P* < .001	4.823(2.561‐9.081)	*P* < .001
cLNM	Negative	1		1		1	
	Positive	22.846(16.271‐32.079)	*P* < .001	15.059 (10.810‐20.979)	*P* < .001	7.992(3.888‐16.430)	*P* < .001
Perineural invasion	Negative	1		1		1	
	Positive	4.379(3.234‐5.928)	*P* < .001	4.312 (3.100‐5.997)	*P* < .001	3.740(2.040‐6.858)	*P* < .001
T stage	T1	1		1		1	
	T2	2.229(1.959‐2.537)	*P* < .001	2.186 (1.903‐2.511)	*P* < .001	2.826(1.813‐4.404)	*P* < .001

Abbreviations: OR, odds ratio; CI, confidence interval; CEA, carcinoembryonic antigen; AD, adenocarcinoma; MAD, mucinous adenocarcinoma; SRCC, signet‐ring cell carcinoma; LNM, lymph node metastasis; cLNM, clinical assessment of lymph node metastasis

**TABLE 3:** Multivariable logistic regression model in the training, internal validation and external validation cohorts

		Multivariate logistic regression					
		Training cohort		Internal validation cohort		External validation cohort	
Subgroups		OR (95% CI)	*P* value	OR (95% CI)	*P* value	OR (95% CI)	*P* value
Age at diagnosis	<60	1		1		1	
	≥60	0.655(0.570‐0.753)	*P* < .001	0.652 (0.562‐0.756)	*P* < .001	0.633 (0.457‐0.876)	.006
Tumor site	Right colon	1		1		1	
	Left colon	1.325(1.152‐1.525)	*P* < .001	1.348 (1.161‐1.564)	*P* < .001	1.939 (1.137‐3.306)	.015
pre‐CEA	Negative	1		1		1	
	Positive	1.283(1.041‐1.580)	.019	1.145 (1.002‐1.424)	.042	2.379 (1.711‐3.309)	*P* < .001
	Other	0.777(0.675‐0.896)	.001	0.752 (0.647‐0.875)	*P* < .001	—	—
Histological type	AD	1		—		—	
	MAD and SRCC	1.304(0.979‐1.738)	.070	—	—	—	—
Tumor grade	I	1		1		1	
	II	1.744(1.382‐2.200)	*P* < .001	1.199 (1.061‐1.495)	.031	1.989 (1.124‐3.523)	.018
	III‐IV	4.445(3.367‐5.870)	*P* < .001	2.359 (1.778‐3.130)	*P* < .001	3.649 (1.861‐7.154)	*P* < .001
cLNM	Negative	1		1		1	
	Positive	18.081(12.736‐25.670)	*P* < .001	12.381 (8.798‐17.422)	*P* < .001	7.339 (3.431‐15.698)	*P* < .001
Perineural invasion	Negative	1		1		1	
	Positive	3.566(2.571‐4.947)	*P* < .001	3.503 (2.460‐4.987)	*P* < .001	2.519 (1.315‐4.827)	.005
T stage	T1	1		1		1	
	T2	1.899(1.650‐2.185)	*P* < .001	1.992 (1.715‐2.313)	*P* < .001	2.068 (1.297‐3.296)	.002

Abbreviations: OR, odds ratio; CI, confidence interval; CEA, carcinoembryonic antigen; LNM, lymph node metastasis; cLNM, clinical assessment of lymph node metastasis.

### 3.3 Evaluation and external validation of the LNM prediction nomogram

**FIGURE 4:**
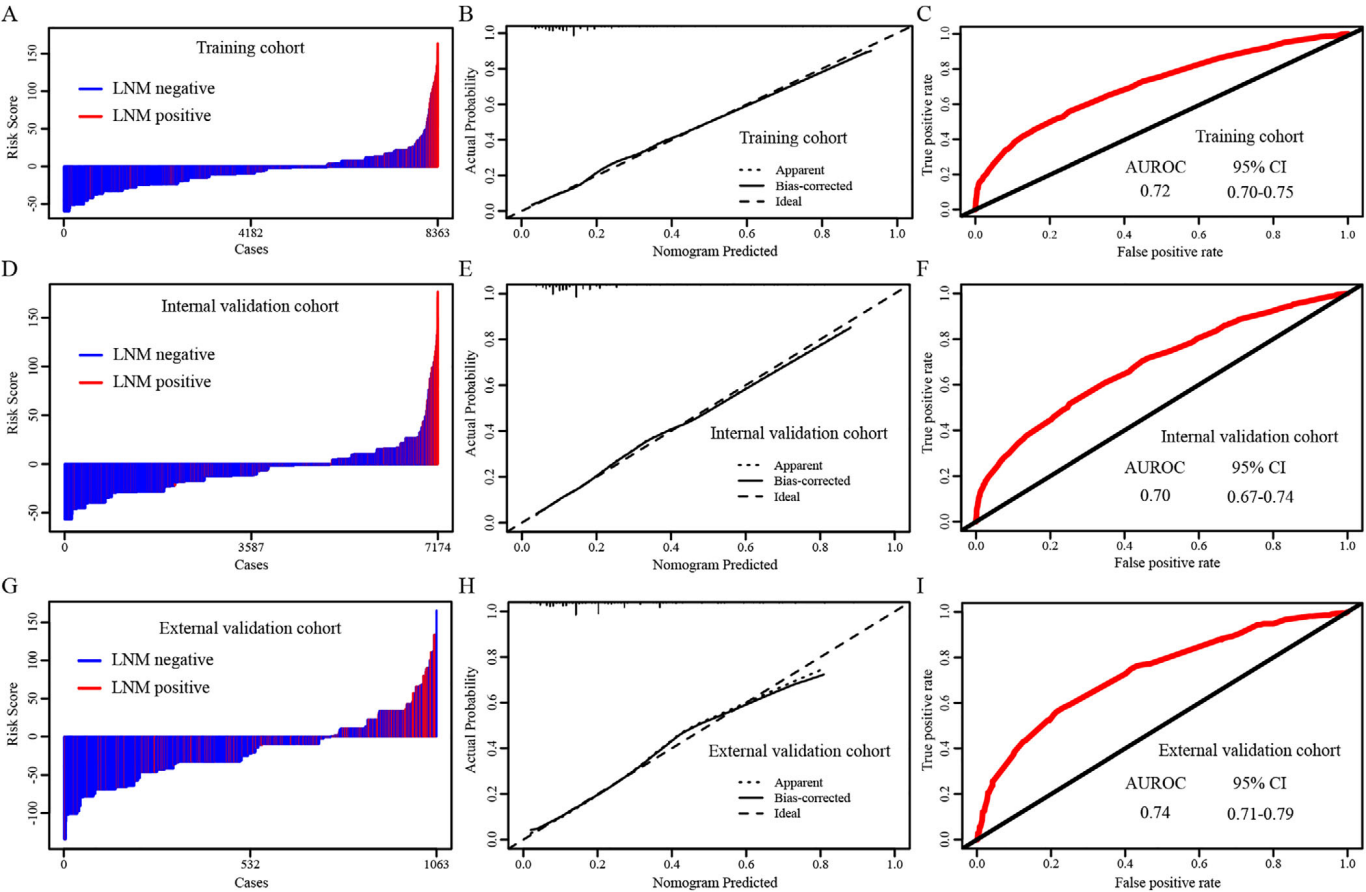
Distribution of risk score and LNM status of T1‐2 CRC patients in the **(A)** training, **(D)** internal validation, and **(G)** external validation cohorts. The calibration curve for predicting LNM of T1‐2 CRC patients in the **(B)** training, **(E)** internal validation, and **(H)** external validation cohorts. AUC values of ROC for predicting LNM of T1‐2 CRC patients in the **(C)** training, **(F)** internal validation, and **(I)** external validation cohorts

The distribution of risk scores and LNM status of the training, internal validation and external validation cohorts are shown in Figures 4A, 4D, and 4G, respectively, suggesting that patients with higher risk scores tend to have LNM positivity. The calibration curve of nomogram is highly consistent with the standard curve, which means high reliability of nomogram prediction ability (Figures 4B, 4D, and 4H). The discrimination ability of the nomogram was represented by the ROC curve. In order to compare the accuracy of the nomogram and cLNM in predicting LNM in T1‐2 CRC patients, logistic ROC analyses on LNM were conducted. The AUROCs of the nomogram for the prediction of LNM were 0.72 (95% confidence interval [CI]: 0.70‐0.75; Figure 4C), 0.70 (95% CI: 0.67‐0.74; Figure 4F), and 0.74 (95% CI: 0.71‐0.79; Figure 4I) in the training, internal validation, and external validation cohorts, respectively, compared with 0.56 (95% CI: 0.52‐0.60; Supporting Information Figure 3A), 0.55 (95% CI: 0.51‐0.58; Supporting Information Figure 3B), and 0.54 (95% CI: 0.50‐0.58; Supporting Information Figure 3C) for cLNM. The sensitivity, specificity, PPV, and NPV of the nomogram in the training cohort were 65.9%, 74.7%, 26.8%, and 91.1%, respectively. In the internal validation cohort, a sensitivity of 61.6%, a specificity of 74.9%, a PPV of 25.4%, and an NPV of 90.3% were detected. In the external validation cohort, a sensitivity of 66.1%, a specificity of 78.6%, a PPV of 39.7%, and an NPV of 87.6% were also found. Moreover, compared with the other six factors, the nomogram showed the best clinical predictive discrimination ability, no matter in training, internal validation or external validation cohorts (Supporting Information Figure 3).

The clinically and mathematically significant predictive performance of the nomogram was validated by stratified analysis. After reclassifying all T1‐2 CRC patients in the SEER and FUSCC cohorts into T1, T2, and cLNM negative subgroups based on T stage and cLNM status, we plotted the logistic ROC curve and distribution of risk scores and LNM status in the three subgroups, which indicated that the nomogram was a beneficial and statistical risk prediction model for the T1, T2, and cLNM negative subgroups (Supporting Information Figures 4 and 5). For all T1 CRC patients, the nomogram yielded an AUROC of 0.68 (95% CI: 0.66‐0.72), 0.66 (95% CI: 0.64‐0.70), and 0.71 (95% CI: 0.68‐0.75) in the training, internal validation, and external validation cohorts, respectively, indicating that the nomogram had favorable discrimination in T1 patients (Supporting Information Figure 4). In terms of all T2 patients, good discrimination ability was also observed among the training (AUROC: 0.70; 95% CI: 0.67‐0.73), internal validation (AUROC: 0.67; 95% CI: 0.65‐0.70), and external validation (AUROC: 0.72; 95% CI: 0.69‐0.76) cohorts (Supporting Information Figure 4). Similar findings were confirmed for the cLNM‐negative subgroup patients in the training, internal validation, and external validation cohorts, with AUROCs of 0.68 (95% CI: 0.65‐0.71), 0.66 (95% CI: 0.63‐0.70), and 0.72 (95% CI: 0.70‐0.75), respectively (Supporting Information Figure 5).

### 3.4 Clinical value of the nomogram

**FIGURE 5:**
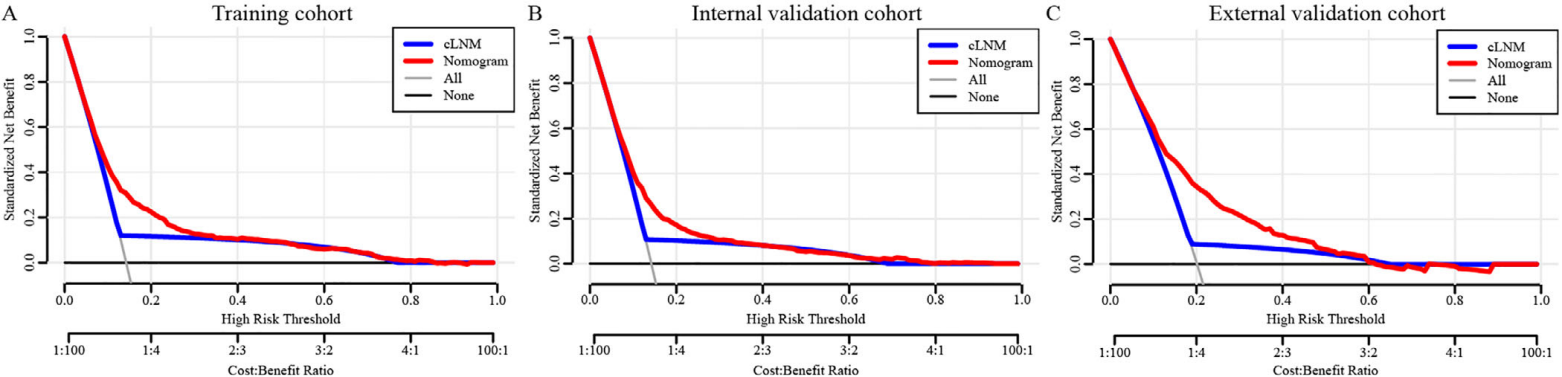
Decision curve analysis of the nomogram and cLNM for predicting LNM of T1‐2 CRC patients in the **(A)** training, **(B)** internal validation, and **(C)** external validation cohorts. The gray line and black line represent the assumption regarding all patients with and without LNM, respectively. The red line represents the nomogram, and the blue line represents the cLNM

The DCA is a new strategy for evaluating alternative predictive treatment methods and has advantages over AUROC in clinical value evaluation. The DCA curves for the developed nomogram and cLNM in the training, internal validation, and external validation cohorts are shown in Figure 5. The black line indicates that no patient had LNM, and the gray line indicates that all patients had LNM. Compared with cLNM, DCA of the nomogram showed higher net benefits, indicating that it had better clinical outcome values than cLNM. Detailed standardized net benefits using the nomogram for specific optimal thresholds are listed in Supporting Information Table 3. By dividing patients in the SEER and FUSCC cohorts into T1 and T2 subgroups, subgroup DCAs indicated that the nomogram had better clinical functional values than cLNM for CRC patients (Supporting Information Figure 6). Furthermore, DCA of the nomogram confirmed high net benefits in not only the entire cLNM‐negative subgroup (Supporting Information Figure 7) but also the T1 and T2 subsets in the cLNM‐negative subgroup (Supporting Information Figure 8), suggesting that it has better clinical application value in the cLNM‐negative subgroup.

## 4 DISCUSSION

The accurate assessment of LNM status is critical for tailored therapy in T1‐2 CRC. In this study, a nomogram incorporating clinical and pathological information in parametric quantities was built to evaluate LNM in T1‐2 CRC patients individually. The characteristics of the nomogram were confirmed regarding its identification and calibration, which contributed to a wide range of applications. The calibration curve and ROC curve showed that the possibility of LNM predicted by the nomogram is highly consistent with the actual LNM status. Moreover, the DCA curve indicated that the nomogram could obtain more potential clinical benefits than cLNM in clinical practice for evaluation of LNM in T1‐2 CRC.

According to current guidelines, whether additional radical surgery plus regional lymphadenectomy should be performed after endoscopic resection of T1 CRC remains controversial. If endoscopic resection is not oncologically safe, which is dictated to a large degree by the probability of LNM, the patient might demand additional surgery to undergo lymphadenectomy.^12^ At present, it is controversial whether T2 CRC is suitable for endoscopic resection, even with endoscopic submucosal dissection techniques. However, recent advances in endoscopic surgery have shown the potential of T2 cancer and endoscopic resection. Such an analysis of the risk and safety factors for LNM in T2 cancer plays an extremely important role in determining whether additional radical surgery plus regional lymphadenectomy is indispensable after endoscopic resection.^13^ Current studies have shown that only 14% of the patients who accepting endoscopic resection of T1 CRC at the outset, were eventually diagnosed as LNM positive after additional radical surgery plus lymphadenectomy.^12^ In other words, over 80% of T1 CRC patients underwent unnecessary additional surgery after endoscopic resection, and some patients even experienced various postoperative complications. For instance, it is necessary in the operation of low rectal carcinoma to perform anus resection and reconstruct an artificial anus when patients undergo additional radical surgery plus lymphadenectomy after endoscopic resection of T1 CRC, which is undoubtedly a great damage to the quality of life for this group. This overtreatment may be due to the lack of current methods to predict LNM risk in patients with T1‐2 CRC. In this study, the sensitivity, specificity, PPV, and NPV of the nomogram were 60%‐70%, 70%‐80%, 25%‐40%, and 85%‐95%, respectively. Compared with cLNM, which is currently used to evaluate LNM status, the sensitivity, specificity, PPV, and NPV of the novel nomogram were significantly improved, which means that it could accurately improve patient survival by accurately predicting LNM to make strategic surgical and adjuvant CT decisions.

In fact, this study showed that patients with different LNM statuses and LNM‐positive patients with or without CT had significantly different survival rates. Randomized clinical trials have shown that stage III patients receiving postoperative adjuvant CT had a survival advantage over those receiving surgery alone.^14^, ^15^ To shorten the time of adjuvant CT and reduce toxicity without losing efficacy, clinical trials began to explore the benefit comparison of 3 versus 6 months of adjuvant CT. According to the results of the IDEA international collaboration, if mFOLFOX6 is selected, T4 and/or N2 colon cancer patients need 6 months of CT to minimize the risk of recurrence. There was no significant correlation between the absolute difference of 3‐year DFS rate between 3 and 6 months of treatment (2%) for the subgroups of T1‐3N1 colon cancer patients.^16^ Therefore, for T1‐2 CRC patients with LNM, if N2 is diagnosed, the 6‐month adjuvant CT is more appropriate, and if N1 is diagnosed, the clinical benefits of 3 months or 6 months of adjuvant CT may be consistent.

At present, the diagnosis of young CRC patients has increased. Relevant research has demonstrated that age was an independent predictive factor of LNM in T1‐2 CRC patients, with younger age associated with more promising outcomes.^17^, ^18^ Xu et al.^18^ argued that the risk of LNM in patients aged 65–79 and over 80 years decreased to approximately 0.65 and 0.44, respectively, compared with patients under 49 years old. It has been reported that lymph node yield decreases with age in CRC patients. In general, the average lymph node yield decreases by 1 for every 7‐year increase in age.^19^ Furthermore, an eventful prognostic factor substantiated by studies was CEA, which is an ideal biomarker for CRC patients.^20^, ^21^, ^22^ Predictable pre‐CEA recording was introduced to monitor LNM. As nomograms have been developed, T1‐2 CRC patients with positive pre‐CEA tended to have significantly higher LNM feasibilities. In addition, left CRC and right CRC were indicated to have different embryological origins.^23^ Diverse features, such as anatomical morphology, immune microenvironment, and molecular pathological characteristics, exist. A previous study associated malignant tumor sites with LNM in CRC patients.^24^ Patients with left CRC had a notably higher rate of LNM, which was also supposed by this research. In addition to age at diagnosis, pre‐CEA level, and tumor location, preceding studies have also shown that histological grade and T stage were identified as independent risk factors for LNM in T1‐2 CRC patients.^25^, ^26^ Histological differentiation was defined as a significant trait to evaluate the advantage of adjuvant chemotherapy in relevant research.^27^ This nomogram verified that poor histological differentiation, for instance signet ring cell carcinoma, was correlated with a worse LNM status. Poor histological grade was considered an unfavorable histopathological feature associated with the adverse clinical course of T1‐2 CRC. The results of this investigation showed that poor histological grade was strongly suspected to give rise to LNM. A higher T stage, T2, was associated with deeper infiltration, which might result in malignant tumor cells transferring into lymph vessels.

In this filed, much work on the prognostic factors and LNM status of CRC has been reported recently. A few researchers reported that their nomogram scoring systems had exceptional capabilities in predicting the LNM and survival of CRC patients.^7^, ^28^ Previous studies on LNM prediction in CRC patients have shown that the clinical advantage of preoperative individualized prediction of LNM in CRC could be enhanced by combining clinical molecular pathological factors and radiomics characteristics, which was proposed to benefit patient OS.^29^ Ozawa et al.^30^ identified several microRNAs, such as MIR32, MIR181B, MIR193B, MIR195, and MIR 411, as promising predictors for LNM in T1 CRC patients. The papers mentioned above were dedicated to predicting the preoperative or postoperative situations of patients, and both might ameliorate the prognosis of patients. However, quite a few studies proposed inspection patterns that had greater traumas or economic burdens to patients. Other studies have not included the results of T1 and T2 CRC patients from multiple centers for verification, and the reliability of these models needs further discussion.

However, this research still has some limitations. First, therapy information except for surgery, such as specific radiotherapy and chemotherapy therapeutics, was not available in the SEER database to be incorporated into the analysis. Second, this was a retrospective study based on limited clinical records and hence was not free from potential selection bias. In addition, the model needs further prospective multicenter clinical research to prove its clinical effectiveness.

## 5 CONCLUSION

In conclusion, we developed and validated a nomogram for predicting the LNM probability of T1‐2 CRC patients. This novel nomogram had sufficient discrimination and calibration capabilities, in addition to exceptional clinical effectiveness, and could be a convenient‐to‐use tool for clinicians to select treatment strategies for patients with T1‐2 CRC.

## AUTHOR CONTRIBUTIONS

SBM, ZZ, and WXD had the idea for this study. WQX and LYH supervised the acquisition of the data. SBM, ZZ, and WXD undertook the statistical analysis. QGL, RJW, and LZ provided statistical advice. All authors contributed to interpretation of the results. SBM and ZZ wrote the article. QGL, SJC, and GXC revised the article and other authors contributed to the content. All authors approved the final version of the manuscript, including the authorship list.

## CONFLICT OF INTEREST

The authors declare that they have no competing interests.

## ETHICS APPROVAL AND CONSENT TO PARTICIPATE

The Ethical Committee and Institutional Review Board of the Fudan University Shanghai Cancer Center reviewed and approved this study protocol. All patients signed written informed consent.

## Supporting information


**Additional file 1: *Supplementary Figure 1***: Kaplan‐Meier survival analysis in all patients. (A) Cause‐specific survival of all patients in the SEER cohort. (B) Disease‐free survival of all patients in the FUSCC cohort. (C) Overall survival of all patients in the FUSCC cohort. ***Supplementary Figure 2***: Kaplan‐Meier analysis of cause‐specific survival (CSS), disease‐free survival (DFS) and overall survival (OS) according to LNM and adjuvant chemotherapy (CT) status. (A) CSS between LNM‐negative and LNM‐positive subgroups in the SEER cohort. (B) CSS among LNM‐negative, LNM‐positive with CT, and LNM‐positive without CT subgroups in the SEER cohort. (C) DFS between LNM‐negative and LNM‐positive subgroups in the FUSCC cohort. (D) DFS among LNM‐negative, LNM‐positive with CT, and LNM‐positive without CT subgroups in the FUSCC cohort. (E) OS between LNM‐negative and LNM‐positive subgroups in the FUSCC cohort. (F) OS among LNM‐negative, LNM‐positive with CT, and LNM‐positive without CT subgroups in the FUSCC cohort. ***Supplementary Figure 3***: Discriminative ability of the seven‐independent risk clinicopathologic characteristics to LNM status in the (A) training, (B) internal validation, and (C) external validation cohorts. ***Supplementary Figure 4***: Subgroup analyses of the nomogram in different T stages. Performance of the nomogram to predict LNM in different T stages of the (A‐D) training, (E‐H) internal validation, and (I‐L) external validation cohorts. ***Supplementary Figure 5***: Subgroup analyses of the nomogram in cLNM‐negative subgroup. Performance of the nomogram to predict LNM in cLNM‐negative subgroup of the (A and B) training, (C and D) internal validation, and (E and F) external validation cohorts. ***Supplementary Figure 6***: Decision curve analysis in different T stages of the (A and B) training, (C and D) internal validation, and (E and F) external validation cohorts. The gray line and black line represent the assumption regarding all patients with and without LNM, respectively. The red line represents the nomogram, and the blue line represents the cLNM. ***Supplementary Figure 7***: Decision curve analysis in cLNM‐negative subgroup of the (A) training, (B) internal validation, and (C) external validation cohorts. The gray line and black line represent the assumption regarding all patients with and without LNM, respectively. The red line represents the nomogram. ***Supplementary Figure 8***: Decision curve analysis in different T stages in cLNM‐negative subgroup of the (A and B) training, (C and D) internal validation, and (E and F) external validation cohorts. The gray line and black line represent the assumption regarding all patients with and without LNM, respectively. The red line represents the nomogram.

Additional **file 2: *Supplementary Table 1***. Comparison of clinicopathological characteristics in the training, internal validation and external validation cohorts (N(%)). ***Supplementary Table 2***. Point assignments and predictive scores for each variable in the nomogram model. ***Supplementary Table 3***. Standardized net benefit using the nomogram for specific optimal thresholds.

## Data Availability

The dataset used during the study are available from the corresponding author on a reasonable request.
