# Supplementary material for: Development and external validation of a predictive scoring system associated with metastasis of T1‐2 colorectal tumors to lymph nodes
Source: Clin Transl Med. 2020 Apr 30;10(1):275–87. doi: 10.1002/ctm2.30 (PMC7240869; doi:10.1002/ctm2.30)
Supplement: Supplementary file 1 — Additional file 1: Supplementary Figure 1: Kaplan‐Meier survival analysis in all patients. (A) Cause‐specific survival of all patients in the SEER cohort. (B) Disease‐free survival of all patients in the FUSCC cohort. (C) Overall survival of all patients in the FUSCC cohort. Supplementary Figure 2: Kaplan‐Meier analysis of cause‐specific survival (CSS), disease‐free survival (DFS) and overall survival (OS) according to LNM and adjuvant chemotherapy (CT) status. (A) CSS between LNM‐negative and LNM‐positive subgroups in the SEER cohort. (B) CSS among LNM‐negative, LNM‐positive with CT, and LNM‐positive without CT subgroups in the SEER cohort. (C) DFS between LNM‐negative and LNM‐positive subgroups in the FUSCC cohort. (D) DFS among LNM‐negative, LNM‐positive with CT, and LNM‐positive without CT subgroups in the FUSCC cohort. (E) OS between LNM‐negative and LNM‐positive subgroups in the FUSCC cohort. (F) OS among LNM‐negative, LNM‐positive with CT, and LNM‐positive without CT subgroups in the FUSCC cohort. Supplementary Figure 3: Discriminative ability of the seven‐independent risk clinicopathologic characteristics to LNM status in the (A) training, (B) internal validation, and (C) external validation cohorts. Supplementary Figure 4: Subgroup analyses of the nomogram in different T stages. Performance of the nomogram to predict LNM in different T stages of the (A‐D) training, (E‐H) internal validation, and (I‐L) external validation cohorts. Supplementary Figure 5: Subgroup analyses of the nomogram in cLNM‐negative subgroup. Performance of the nomogram to predict LNM in cLNM‐negative subgroup of the (A and B) training, (C and D) internal validation, and (E and F) external validation cohorts. Supplementary Figure 6: Decision curve analysis in different T stages of the (A and B) training, (C and D) internal validation, and (E and F) external validation cohorts. The gray line and black line represent the assumption regarding all patients with and without LNM, respectively [file CTM2-10-275-s001.docx]

**Additional file 1:**


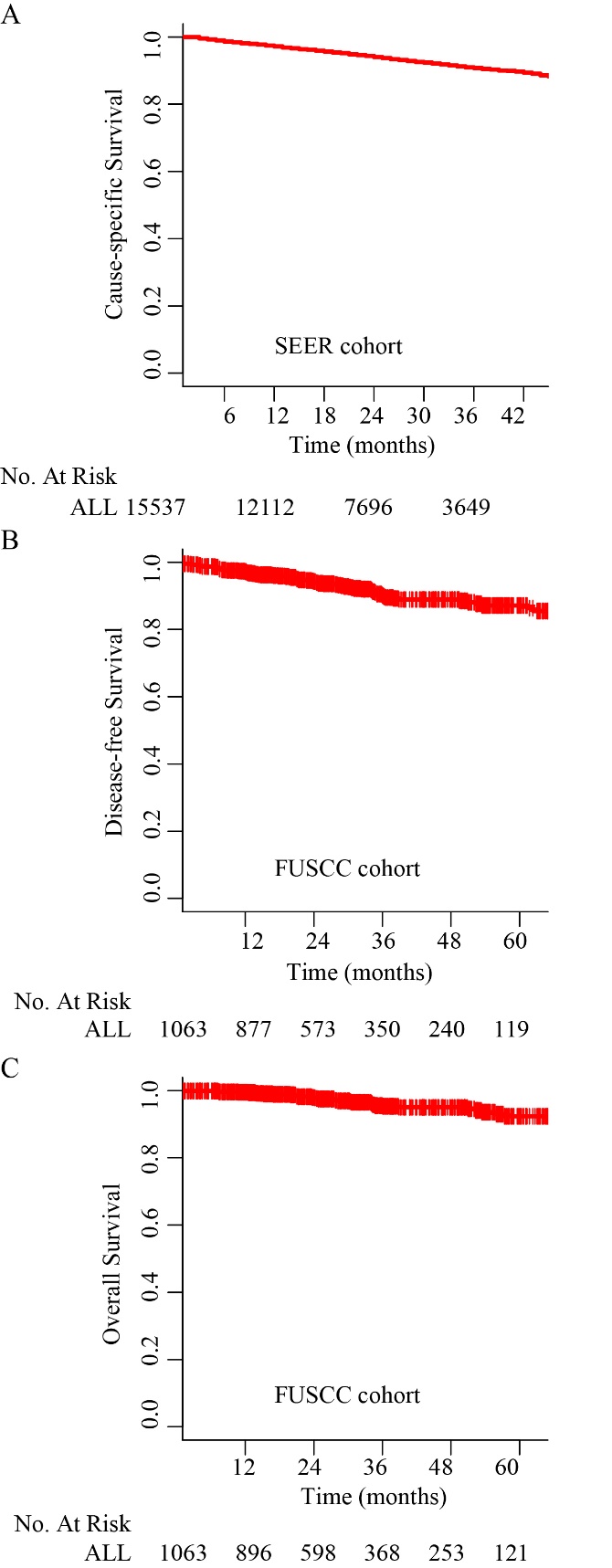


**Supplementary Figure 1:** Kaplan-Meier survival analysis in all patients. (A) Cause-specific survival of all patients in the SEER cohort. (B) Disease-free survival of all patients in the FUSCC cohort. (C) Overall survival of all patients in the FUSCC cohort.


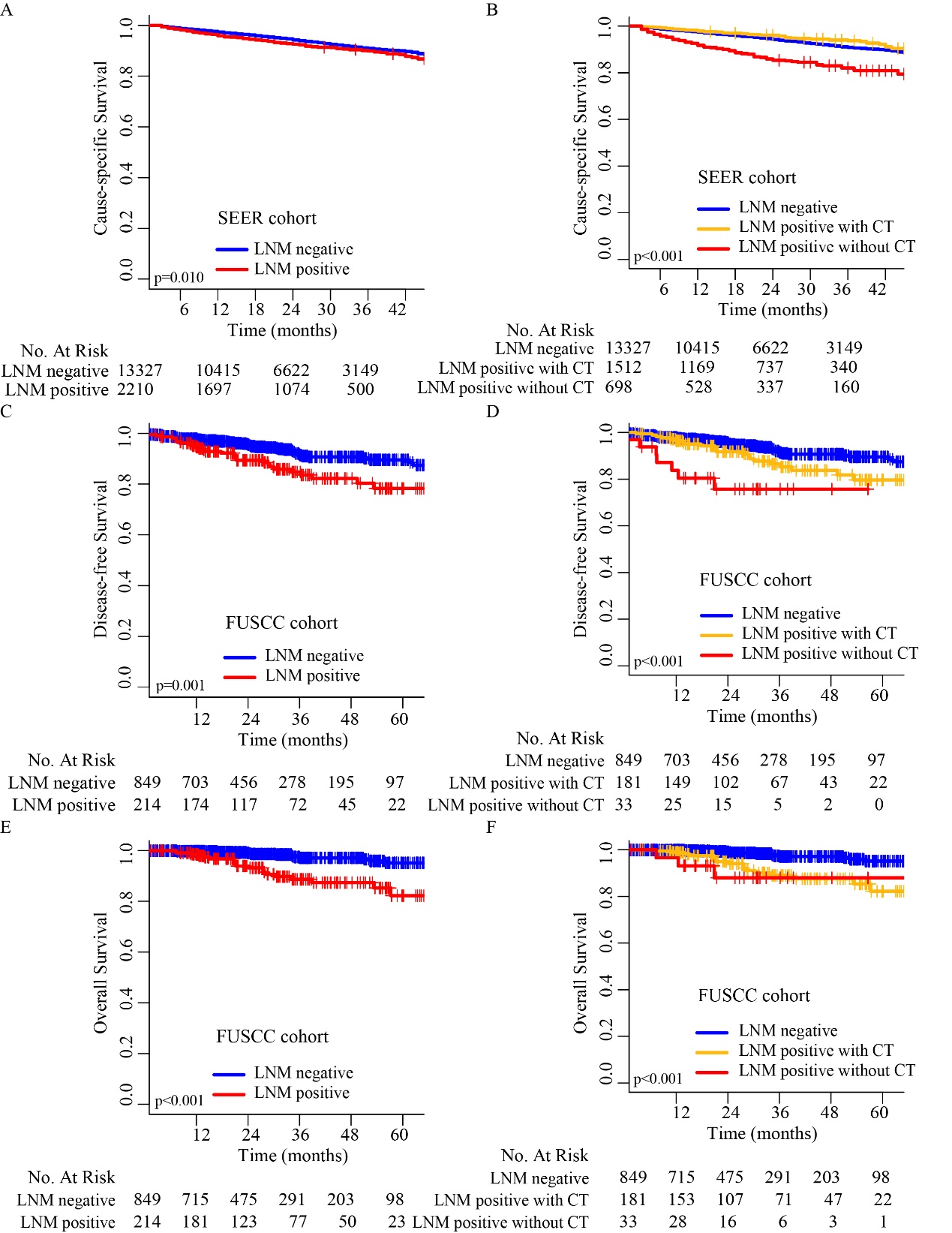


**Supplementary Figure 2:** Kaplan-Meier analysis of cause-specific survival (CSS), disease-free survival (DFS) and overall survival (OS) according to LNM and adjuvant chemotherapy (CT) status. (A) CSS between LNM-negative and LNM-positive subgroups in the SEER cohort. (B) CSS among LNM-negative, LNM-positive with CT, and LNM-positive without CT subgroups in the SEER cohort. (C) DFS between LNM-negative and LNM-positive subgroups in the FUSCC cohort. (D) DFS among LNM-negative, LNM-positive with CT, and LNM-positive without CT subgroups in the FUSCC cohort. (E) OS between LNM-negative and LNM-positive subgroups in the FUSCC cohort. (F) OS among LNM-negative, LNM-positive with CT, and LNM-positive without CT subgroups in the FUSCC cohort.


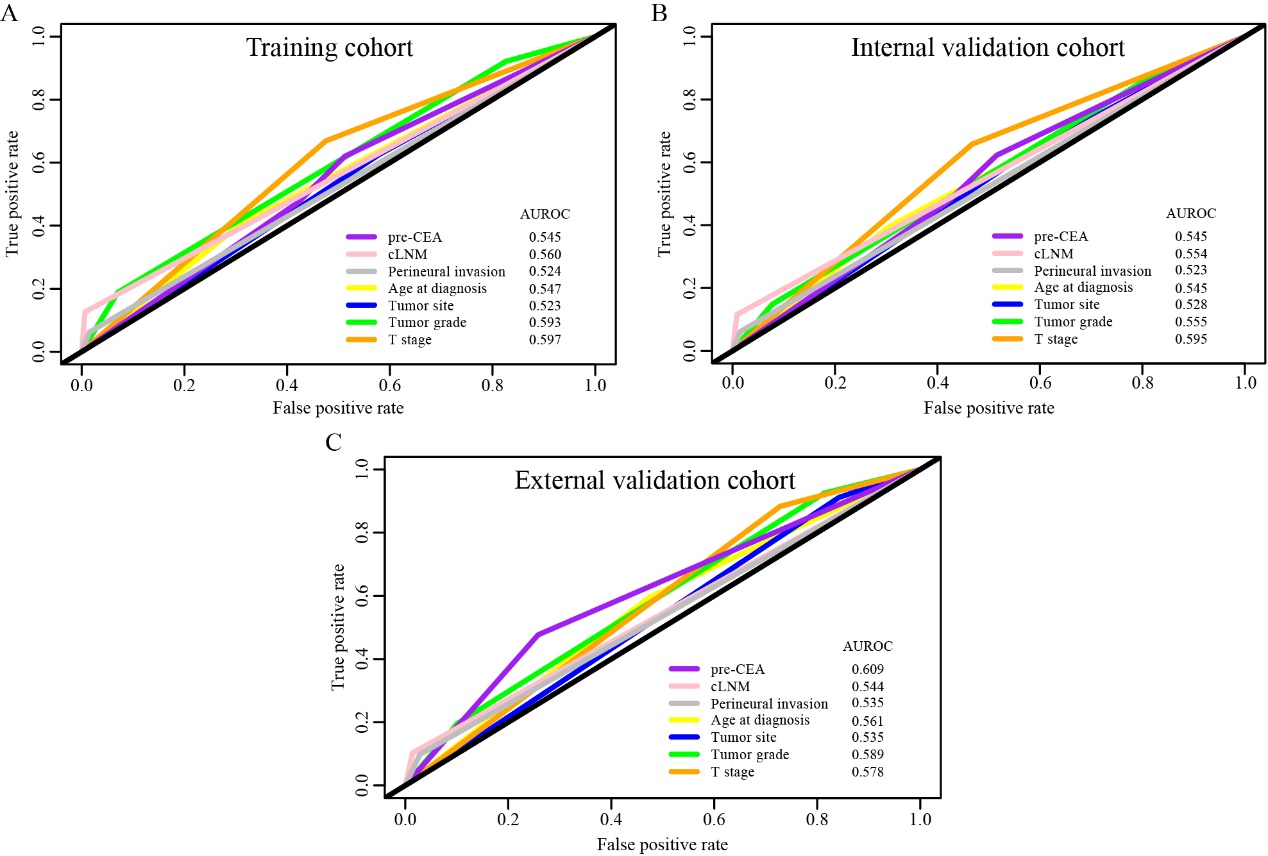


**Supplementary Figure 3:** Discriminative ability of the seven-independent risk clinicopathologic characteristics to LNM status in the (A) training, (B) internal validation, and (C) external validation cohorts.


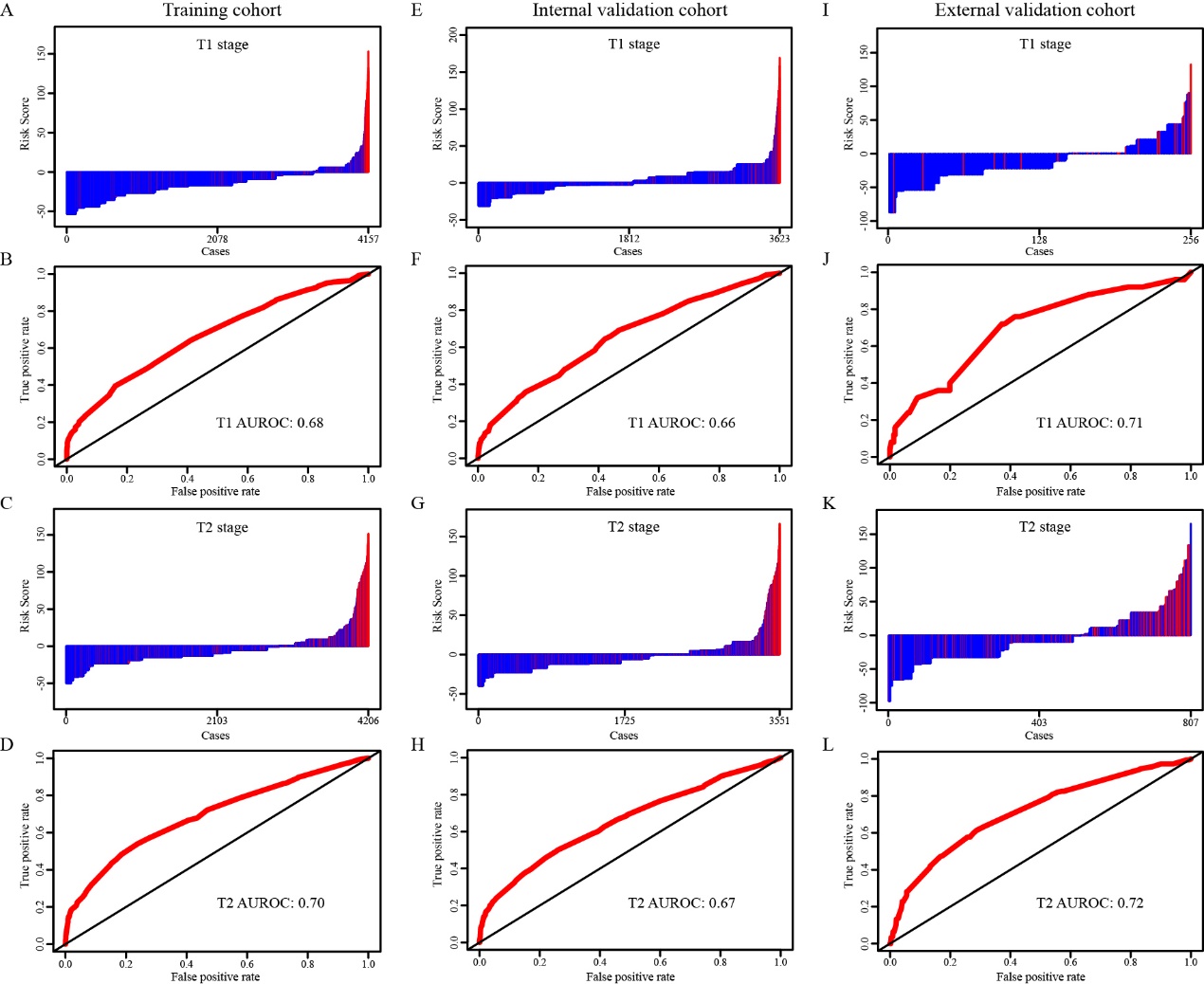


**Supplementary Figure 4:** Subgroup analyses of the nomogram in different T stages. Performance of the nomogram to predict LNM in different T stages of the (A-D) training, (E-H) internal validation, and (I-L) external validation cohorts.


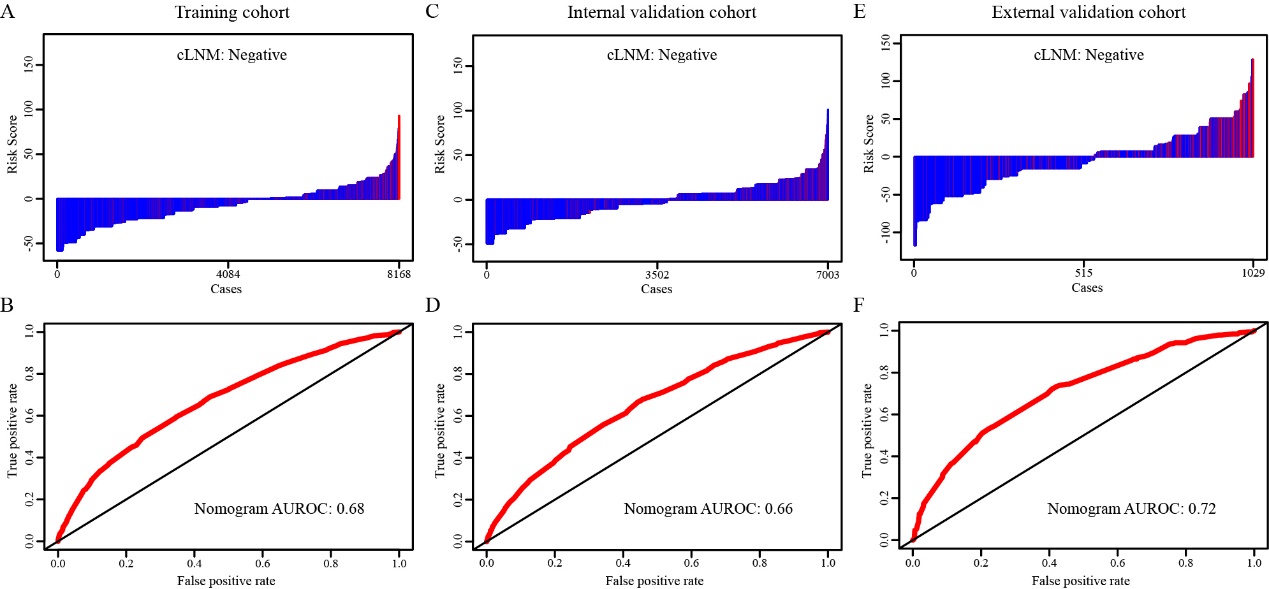


**Supplementary Figure 5:** Subgroup analyses of the nomogram in cLNM-negative subgroup. Performance of the nomogram to predict LNM in cLNM-negative subgroup of the (A and B) training, (C and D) internal validation, and (E and F) external validation cohorts.


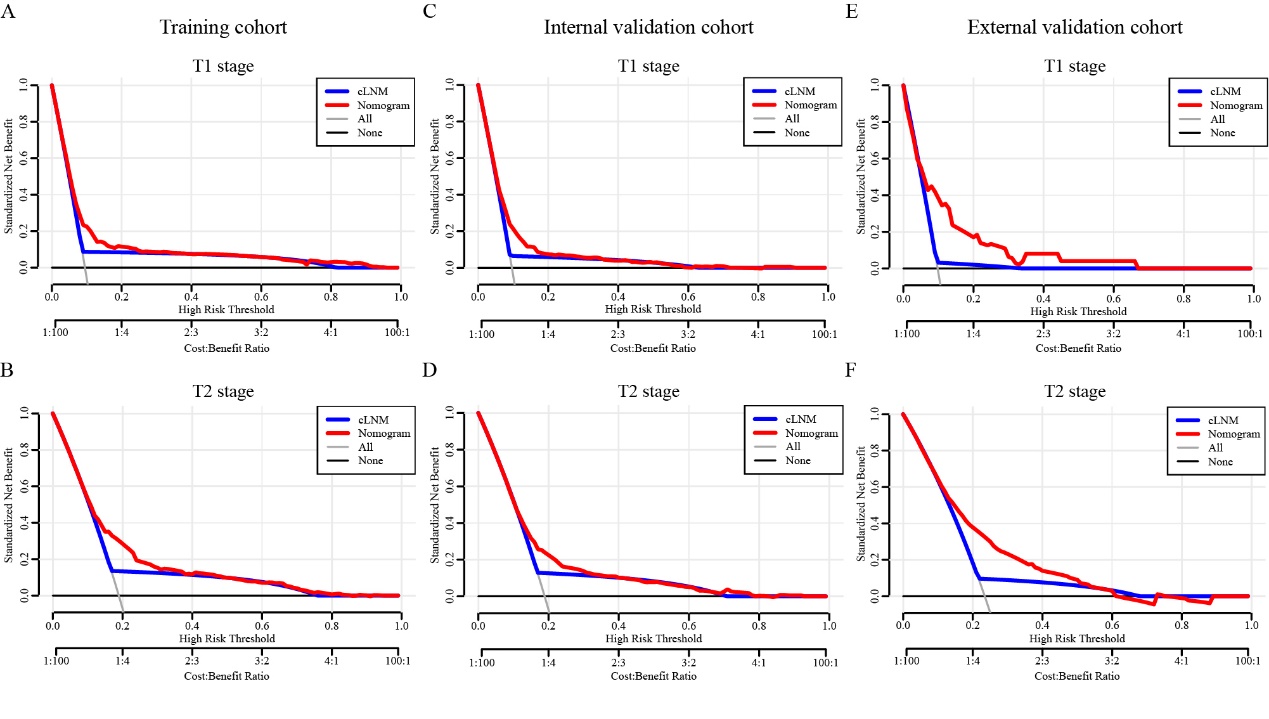


**Supplementary Figure 6:** Decision curve analysis in different T stages of the (A and B) training, (C and D) internal validation, and (E and F) external validation cohorts. The gray line and black line represent the assumption regarding all patients with and without LNM, respectively. The red line represents the nomogram, and the blue line represents the cLNM.


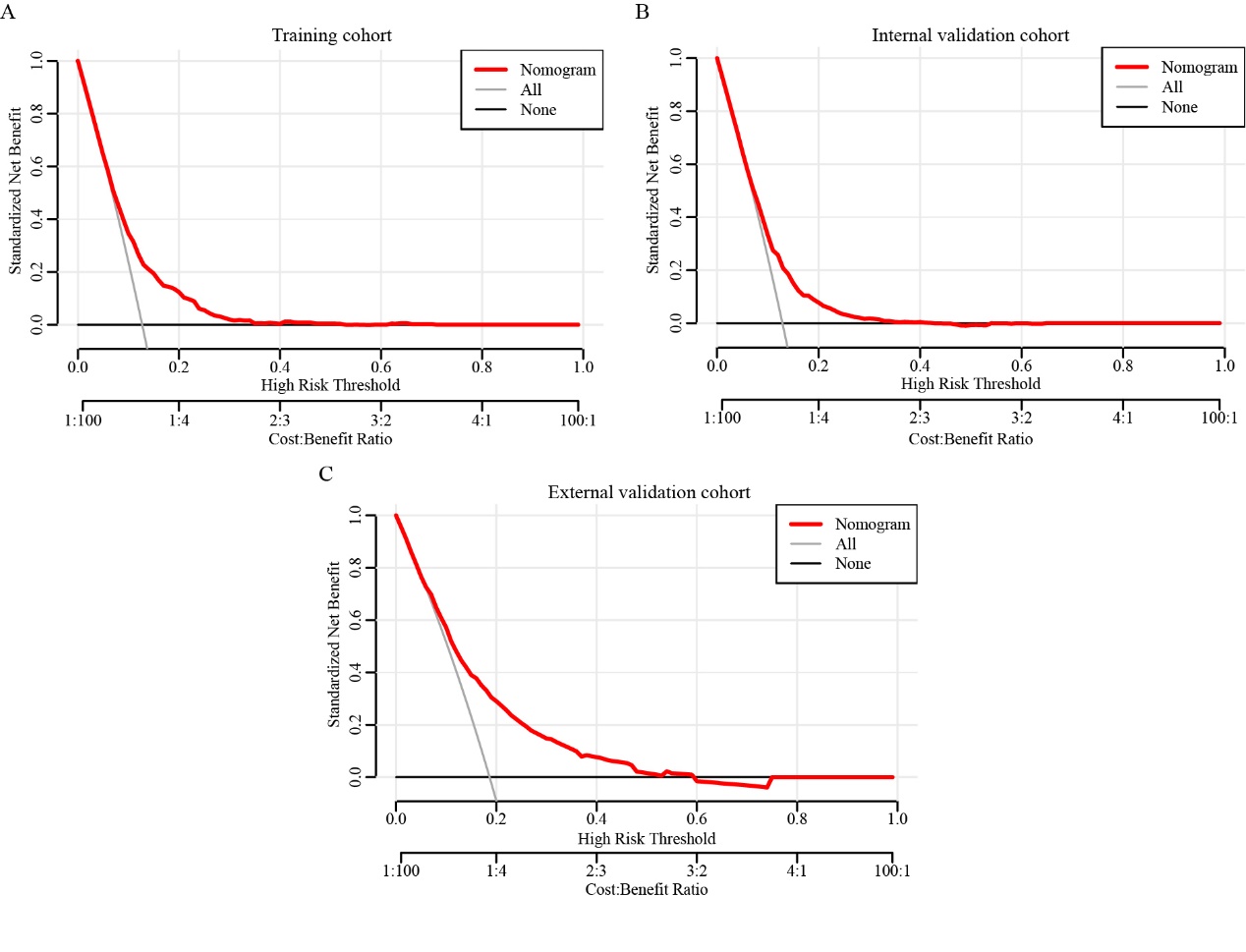


**Supplementary Figure 7:** Decision curve analysis in cLNM-negative subgroup of the (A) training, (B) internal validation, and (C) external validation cohorts. The gray line and black line represent the assumption regarding all patients with and without LNM, respectively. The red line represents the nomogram.


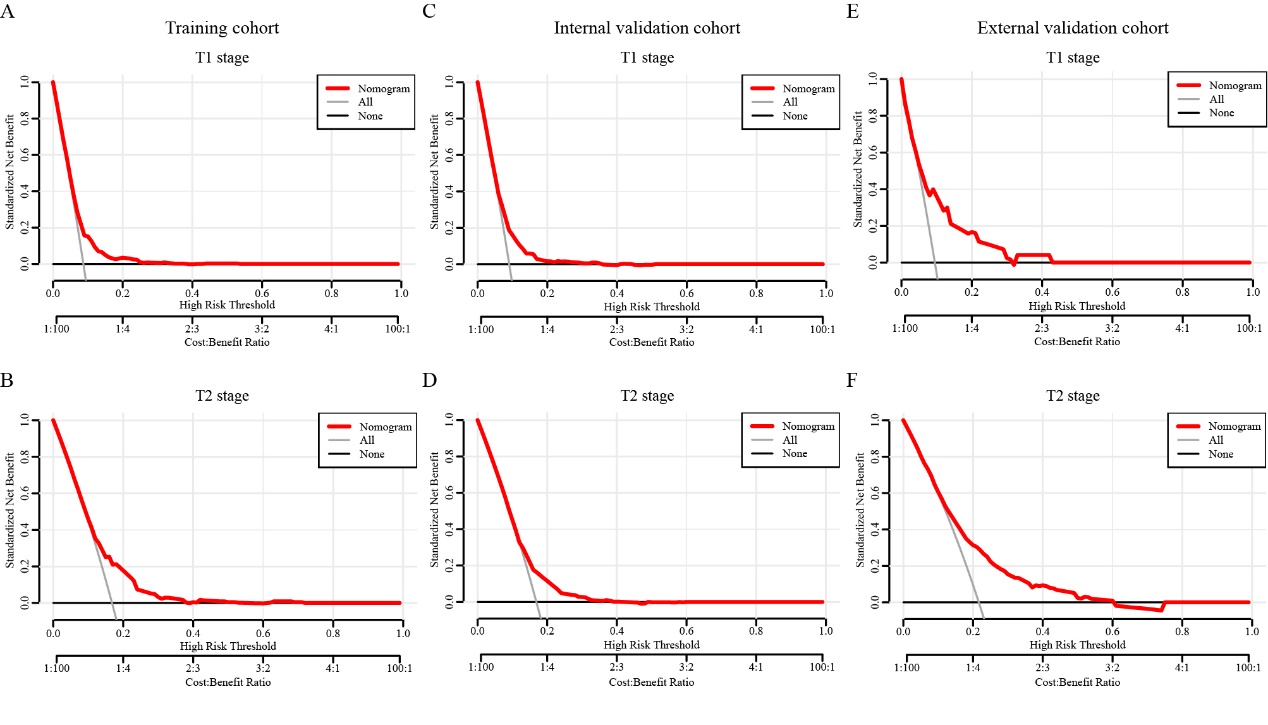


**Supplementary Figure 8:** Decision curve analysis in different T stages in cLNM-negative subgroup of the (A and B) training, (C and D) internal validation, and (E and F) external validation cohorts. The gray line and black line represent the assumption regarding all patients with and without LNM, respectively. The red line represents the nomogram.
