# Supplementary material for: Development and external validation of a predictive scoring system associated with metastasis of T1‐2 colorectal tumors to lymph nodes
Source: Clin Transl Med. 2020 Apr 30;10(1):275–87. doi: 10.1002/ctm2.30 (PMC7240869; doi:10.1002/ctm2.30)
Supplement: Supplementary file 2 — Additional file 2: Supplementary Table 1. Comparison of clinicopathological characteristics in the training, internal validation and external validation cohorts (N(%)). Supplementary Table 2. Point assignments and predictive scores for each variable in the nomogram model. Supplementary Table 3. Standardized net benefit using the nomogram for specific optimal thresholds. [file CTM2-10-275-s002.docx]

| Supplementary Table 1. Comparison of clinicopathological characteristics in the training, internal validation and external validation cohorts (N(%)). | | | | | | | | | |
| --- | --- | --- | --- | --- | --- | --- | --- | --- | --- |
| Characteristics | SEER | | |  | FUSCC |  | p values | | |
|  | Training cohort |  | Internal validation cohort |  | External validation cohort |  | Training cohort |  | Training cohort |
|  | N=8363 |  | N=7174 |  | N=1063 |  | vs. |  | vs. |
|  |  |  |  |  |  |  | Internal validation cohort |  | External validation cohort |
| Age |  |  |  |  |  |  | 0.756 |  | p<0.001 |
| <60 | 2675(32.0) |  | 2278(31.8) |  | 528(49.7) |  |  |  |  |
| ≥60 | 5688(68.0) |  | 4896(68.2) |  | 535(50.3) |  |  |  |  |
| Mean±SD | 66.0±12.9 |  | 65.9±12.6 |  | 59.2±11.4 |  | 0.317 |  | p<0.001 |
| Median (IQR) | 66(56-76) |  | 66(56-75) |  | 60(52-67) |  |  |  |  |
| Gender |  |  |  |  |  |  | 0.583 |  | 0.243 |
| Female | 4077(48.8) |  | 3529(49.2) |  | 498(46.8) |  |  |  |  |
| Male | 4286(51.2) |  | 3645(50.8) |  | 565(53.2) |  |  |  |  |
| Primary site |  |  |  |  |  |  | 0.618 |  | p<0.001 |
| Right | 3463(41.4) |  | 2999(41.8) |  | 153(14.4) |  |  |  |  |
| Left | 4900(58.6) |  | 4175(58.2) |  | 910(85.6) |  |  |  |  |
| Grade |  |  |  |  |  |  | 0.695 |  | 0.001 |
| I | 1344(16.1) |  | 1117(15.6) |  | 174(16.4) |  |  |  |  |
| II | 6291(75.2) |  | 5430(75.7) |  | 761(71.6) |  |  |  |  |
| III and IV | 728(8.7) |  | 627(8.7) |  | 128(12.0) |  |  |  |  |
| Histological type |  |  |  |  |  |  | 0.350 |  | p<0.001 |
| AD | 7978(95.4) |  | 6866(95.7) |  | 986(92.8) |  |  |  |  |
| MAD and SRCC | 385(4.6) |  | 308(4.3) |  | 77(7.2) |  |  |  |  |
| Tumor Size |  |  |  |  |  |  | p<0.001 |  | 0.012 |
| <4 | 5312(63.5) |  | 4758(66.3) |  | 633(59.5) |  |  |  |  |
| ≥4 | 3051(36.5) |  | 2416(33.7) |  | 430(40.5) |  |  |  |  |
| Perineural invasion |  |  |  |  |  |  | 0.892 |  | p<0.001 |
| No | 8182(97.8) |  | 7021(97.9) |  | 1018(95.8) |  |  |  |  |
| Yes | 181(2.2) |  | 153(2.1) |  | 45(4.2) |  |  |  |  |
| Pre-CEA |  |  |  |  |  |  | 0.452 |  | p<0.001 |
| Negative | 3592(43.0) |  | 3060(42.7) |  | 742(69.8) |  |  |  |  |
| Positive | 822(9.8) |  | 749(10.4) |  | 321(30.2) |  |  |  |  |
| Other | 3949(47.2) |  | 3365(46.9) |  | — |  |  |  |  |
| cLNM |  |  |  |  |  |  | 0.832 |  | 0.084 |
| Negative | 8168(97.7) |  | 7003(97.6) |  | 1029(96.8) |  |  |  |  |
| Positive | 195(2.3) |  | 171(2.4) |  | 34(3.2) |  |  |  |  |
| Adjuvant CT |  |  |  |  |  |  | 0.221 |  | p<0.001 |
| No | 7403(88.5) |  | 6305(87.9) |  | 855(80.4) |  |  |  |  |
| Yes | 960(11.5) |  | 869(12.1) |  | 208(19.6) |  |  |  |  |
| T stage |  |  |  |  |  |  | 0.323 |  | p<0.001 |
| T1 | 4157(49.7) |  | 3623(50.5) |  | 256(24.1) |  |  |  |  |
| T2 | 4206(50.3) |  | 3551(49.5) |  | 807(75.9) |  |  |  |  |
| SEER, Surveillance, Epidemiology, and End Results; FUSCC, Fudan University Shanghai Cancer Center; LNM, lymph node metastasis; SD, standard deviation; IQR, interquartile range; CEA, carcinoembryonic antigen; AD, adenocarcinoma; MAD, mucinous adenocarcinoma; SRCC, signet-ring cell carcinoma; CT, chemotherapy | | | | | | | | | |

| Supplementary Table 2. Point assignments and predictive scores for each variable in the nomogram model |
| --- |

| Variables | Classification | Nomogram score |
| --- | --- | --- |
| Age at diagnosis | ≥60 | 0 |
|  | <60 | 13.98 |
| Tumor site | Right | 0 |
|  | Left | 9.51 |
| Tumor grade | I | 0 |
|  | II | 26.97 |
|  | III and IV | 53.94 |
| T stage | T1 | 0 |
|  | T2 | 22.41 |
| pre-CEA | Other | 0 |
|  | Negative | 4.07 |
|  | Positive | 8.14 |
| cLNM | Negative | 0 |
|  | Positive | 100 |
| Perineural invasion | Negative | 0 |
|  | Positive | 43.04 |
| pre-CEA, preoperative carcinoembryonic antigen; cLNM, clinical assessment of lymph node metastasis | | |

| Supplementary Table 3. Standardized net benefit using the nomogram for specific optimal thresholds. | | | | | | | | | | | |
| --- | --- | --- | --- | --- | --- | --- | --- | --- | --- | --- | --- |
| Threshold | Standardized net benefit for all | | |  | Standardized net benefit for cLNM | | |  | Standardized net benefit for nomogram | | |
|  | Training | Internal validation | External validation |  | Training | Internal validation | External validation |  | Training | Internal validation | External validation |
| 0.1 | 0.330 | 0.330 | 0.559 |  | 0.330 | 0.330 | 0.559 |  | 0.424 | 0.403 | 0.610 |
| 0.2 |  |  | 0.008 |  | 0.117 | 0.103 | 0.089 |  | 0.225 | 0.172 | 0.345 |
| 0.3 |  |  |  |  | 0.110 | 0.093 | 0.079 |  | 0.127 | 0.106 | 0.218 |
| 0.4 |  |  |  |  | 0.101 | 0.081 | 0.065 |  | 0.104 | 0.081 | 0.129 |
| 0.5 |  |  |  |  | 0.088 | 0.064 | 0.047 |  | 0.092 | 0.053 | 0.065 |
| 0.6 |  |  |  |  | 0.069 | 0.038 | 0.019 |  | 0.060 | 0.037 | 0.009 |
| 0.7 |  |  |  |  | 0.038 | 0 |  |  | 0.043 | 0.020 | 0 |
| 0.8 |  |  |  |  | 0 |  |  |  | 0.009 | 0.001 |  |
| 0.9 |  |  |  |  |  |  |  |  | 0 | 0.004 |  |
| cLNM, clinical assessment of lymph node metastasis | | | | | | | | | | | |
